# Predictive value of inflammation and nutritional index in immunotherapy for stage IV non-small cell lung cancer and model construction

**DOI:** 10.1038/s41598-024-66813-4

**Published:** 2024-07-30

**Authors:** Wenqian Lei, Wei Wang, Shixiang Qin, Weirong Yao

**Affiliations:** 1 Graduate School, Jiangxi Medical College, Nanchang University, 461 Bayi Avenue, Donghu District, Nanchang, 330006 Jiangxi China; 2 Department of Oncology, Jiangxi Provincial People’s Hospital, 152 Aiguo Road, Nanchan, 330006 Jiangxi China; 3 Postgraduate training base Alliance of Wenzhou Medical University (Zhejiang Cancer Hospital), Wenzhou Medical University, 270 Xueyuanxi Road, Lucheng District, Wenzhou, 325027 Zhejiang China; 4 Graduate School, Bengbu Medical University, Bengbu, 233030 Anhui China

**Keywords:** Immunotherapy, NSCLC, Nomogram model, LAR, PIV, PNI, Cancer, Immunology

## Abstract

Identifying individuals poised to gain from immune checkpoint inhibitor (ICI) therapies is a pivotal element in the realm of tailored healthcare. The expression level of Programmed Death Ligand 1 (PD-L1) has been linked to the response to ICI therapy, but its assessment typically requires substantial tumor tissue, which can be challenging to obtain. In contrast, blood samples are more feasible for clinical application. A number of promising peripheral biomarkers have been proposed to overcome this hurdle. This research aims to evaluate the prognostic utility of the albumin-to-lactate dehydrogenase ratio (LAR), the Pan-immune-inflammation Value (PIV), and the Prognostic Nutritional Index (PNI) in predicting the response to ICI therapy in individuals with advanced non-small cell lung cancer (NSCLC). Furthermore, the study seeks to construct a predictive nomogram that includes these markers to facilitate the selection of patients with a higher likelihood of benefiting from ICI therapy. A research initiative scrutinized the treatment records of 157 advanced NSCLC patients who received ICI therapy across two Jiangxi medical centers. The cohort from Jiangxi Provincial People’s Hospital (comprising 108 patients) was utilized for the training dataset, while the contingent from Jiangxi Cancer Hospital (49 patients) served for validation purposes. Stratification was based on established LAR, PIV, and PNI benchmarks to explore associations with DCR and ORR metrics. Factorial influences on ICI treatment success were discerned through univariate and multivariate Cox regression analysis. Subsequently, a Nomogram was devised to forecast outcomes, its precision gauged by ROC and calibration curves, DCA analysis, and cross-institutional validation. In the training group, the optimal threshold values for LAR, PIV, and PNI were identified as 5.205, 297.49, and 44.6, respectively. Based on these thresholds, LAR, PIV, and PNI were categorized into high (≥ Cut-off) and low (< Cut-off) groups. Patients with low LAR (L-LAR), low PIV (L-PIV), and high PNI (H-PNI) exhibited a higher disease control rate (DCR) (P < 0.05) and longer median progression-free survival (PFS) (P < 0.05). Cox multivariate analysis indicated that PS, malignant pleural effusion, liver metastasis, high PIV (H-PIV), and low PNI (L-PNI) were risk factors adversely affecting the efficacy of immunotherapy (P < 0.05). The Nomogram model predicted a concordance index (C-index) of 0.78 (95% CI: 0.73–0.84). The areas under the ROC curve (AUC) for the training group at 6, 9, and 12 months were 0.900, 0.869, and 0.866, respectively, while the AUCs for the external validation group at the same time points were 0.800, 0.886, and 0.801, respectively. Throughout immunotherapy, PIV and PNI could act as prospective indicators for forecasting treatment success in NSCLC patients, while the devised Nomogram model exhibits strong predictive performance for patient prognoses.

## Introduction

Globally, lung cancer stands as a primary cause of cancer-related mortality, with non-small cell lung cancer (NSCLC) representing about 80–85% of all lung cancer cases^1,2^. Lately, the introduction of immune checkpoint inhibitors (ICIs) that focus on the programmed cell death receptor 1 and its ligand (PD-1/PD-L1) has become a promising therapeutic approach for advanced NSCLC patients without responsive genetic alterations^3^. This has not only prolonged the survival of NSCLC patients but also revolutionized the therapeutic paradigm for NSCLC. However, the efficacy of ICIs is modest in some populations, and the related adverse events, known as immune-related adverse events (irAEs), highlight the limitations of ICIs. Data indicates that the response rate to ICIs is below 20% in unselected patient groups^4^, leading to significant treatment expenses without corresponding benefits and the potential for severe immunotoxic side effects. Consequently, identifying biomarkers that can predict the response to ICI therapy is of paramount importance. Currently, clinical practices commonly utilize immunohistochemistry to evaluate PD-L1 expression levels and assess tumor mutation burden (TMB) in tumor samples, but this approach has its own challenges. These include difficulties in sampling^5^, high costs, tumor microenvironment heterogeneity (with PD-1/PD-L1 expression potentially lower in primary tumors than in metastatic sites^6^), and the inability to dynamically monitor treatment responses (since PD-1/PD-L1 expression can fluctuate over time and with different anti-tumor treatment strategies^6^).

Peripheral blood markers such as lactate dehydrogenase (LDH)^7^, granulocytes, platelets, lymphocytes, and albumin, as well as derived biomarkers, can provide quick and sensitive indications of inflammation, immune response, and nutritional status in the human body, reflecting the progression of tumors in patients. Evidence has shown that the pan-Immune-inflammation value (PIV) is associated with the advancement and prognostic outcomes in several types of cancer, such as colorectal, breast, and melanoma^8–10^. It has recently been shown that the Prognostic Nutritional Index (PNI), which has historically been used to evaluate preoperative nutritional status, can predict the effectiveness of immunotherapy in NSCLC when assessed before to treatment^11^. However, studies on the lactate dehydrogenase-to-albumin ratio (LAR) as a predictor of immunotherapy efficacy in NSCLC are still scarce.

Therefore, this article primarily investigates the prognostic significance of these peripheral blood markers for the efficacy of ICIs and aims to construct a nomogram that integrates baseline characteristics to assess treatment outcomes. This could provide practical and straightforward biomarkers for the personalized and precise treatment of patients with advanced NSCLC.

## Materials and methods

### Patients

A retrospective analysis was carried out on 108 advanced NSCLC patients treated with ICIs treatment from September 2019 to June 2023 at Jiangxi Provincial People's Hospital, along with another group of 49 advanced NSCLC patients who underwent ICI therapy at Jiangxi Cancer Hospital. Inclusion criteria were as follows: (1) age over 18 years; (2) histopathological diagnosis of NSCLC as per the “Guidelines for the Diagnosis and Treatment of Primary Lung Cancer (2022 edition)”; (3) accordance to the AJCC 8th edition TNM staging criteria, categorised as stage VI, with at least one lesion assessable by imaging; (4) completion of at least three cycles of immunotherapy; (5) availability of complete clinical records, blood test results, and radiological reports during the course of ICIs treatment. Exclusion criteria included: (1) concurrent infection or hematologic diseases; (2) development of myelosuppression during treatment; (3) severe cardiac, hepatic, or renal dysfunction.

### Data collection

Patient clinical information was gathered from the electronic health records system, encompassing variables such as sex, age, body mass index (BMI), pathological type, tumor staging, distant metastasis status, treatment regimen, number of treatment lines, and complete peripheral blood indices taken on the day of or within 21 days after the first immunotherapy session. Additionally, indices such as the PIV, PNI, and LAR were calculated. The PIV is calculated using the formula: neutrophil count × platelet count × monocyte count/lymphocyte count. The PNI is determined by the equation: serum albumin (g/L) + 5 × lymphocyte count. The LAR is the ratio of lactate dehydrogenase (LDH) to serum albumin.

### Treatment

Immunotherapy with PD-1/PD-L1 inhibitors is administered as follows:

(1) Sintilimab: 200 mg administered intravenously (IV), every 3 weeks; (2) Tislelizumab: 200 mg IV, every 3 weeks; (3) Toripalimab: 3 mg/kg IV, every 2 weeks; (4) Pembrolizumab: 2 mg/kg IV, every 3 weeks; (5) Nivolumab: 3 mg/kg IV, every 2 weeks; (6) Camrelizumab: 200 mg per dose, every 2 weeks; (7) Atezolizumab: 1200 mg per dose, every 3 weeks. When combined with chemotherapy, ICIs are used in conjunction with platinum-based doublet chemotherapy, with the chemotherapeutic agents selected based on the tumor's histopathological type. These agents include docetaxel, albumin-bound paclitaxel/nab-paclitaxel, gemcitabine, and pemetrexed. The combination of ICIs with anti-angiogenic drugs includes agents such as endostatin, bevacizumab, and anlotinib. Among the patients, some with EGFR mutations are treated with immunotherapy as a later line of therapy post-resistance to previous treatments.

### Efficiency assessment

Based on the RECIST criteria version 1.1^12^, patient outcomes from treatment are categorized into four types: complete response (CR), partial response (PR), stable disease (SD), and progressive disease (PD). The objective response rate (ORR) is calculated as (CR + PR)/(CR + PR + SD + PD) × 100%, and the disease control rate (DCR) is calculated as (CR + PR + SD)/(CR + PR + SD + PD) × 100%. Progression-free survival (PFS) is the duration from the start of treatment with immune checkpoint inhibitors to the point of disease progression, the appearance of an endpoint event, or up to the most recent patient check-in, with the final date for gathering patient data being August 1, 2023.

### Statistical analysis

For our study, we aimed to detect a medium effect size (Cohen’s d = 0.5) with a significance level (α) of 0.05 and a power (1−β) of 0.80. Using G*Power software version 3.1.9.7, the required sample size was calculated to be approximately 100 patients. Our study included 157 advanced NSCLC patients (108 in the training cohort and 49 in the validation cohort), which is sufficient to detect medium effect sizes and draw robust conclusions. Continuous variable indices such as the LAR, PIV, and PNI were analyzed using GraphPad Prism software version 9.5 to calculate cutoff values for dichotomization. The COX proportional hazards model was then applied to perform univariate regression analysis on these baseline indices, and variables showing significance (p < 0.05) were included in a multivariate regression analysis. A Nomogram model was constructed using R language version 3.5.1, and the model’s predictive performance was evaluated by plotting the C-index, calibration curves, Decision Curve Analysis (DCA) curves, and performing external validation.

### Ethics approval and consent to participate

The study was approved by the Medical Ethics Committee of Jiangxi Provincial People's Hospital. All methods were carried out in accordance with relevant guidelines and regulations. All methods were carried out in accordance with declaration of Helsinki. Informed consent was waived by the Jiangxi Provincial People's Hospital due to the retrospective nature of this study. Ethical Approval ID: V1.0,20240209.

## Results

### Patient characteristics and optimal threshold values for LAR, PIV, and PNI

The research encompassed 157 advanced NSCLC patients from two medical institutions. A group of 108 patients from Jiangxi Provincial People’s Hospital constituted the modeling training cohort, while 49 patients from Jiangxi Cancer Hospital were used for external validation. Utilizing GraphPad Prism software, the ideal threshold levels for the modeling training cohort were identified: a LAR of 5.205 (achieving an AUC of 0.740), a PIV of 297.49 (with an AUC of 0.701), and a PNI of 44.6 (with an AUC of 0.781). In the validation group, the thresholds were established at a LAR of 4.311 (with an AUC of 0.690), a PIV of 203.080 (with an AUC of 0.871), and a PNI of 47.525 (with an AUC of 0.739). Based on these optimal cutoff values, patients were categorized into Low-LAR/High-LAR groups, Low-PIV/High-PIV groups, and Low-PNI/High-PNI groups. Furthermore, all variables analyzed between the training and validation cohorts showed no statistical significance (p > 0.05), indicating that there were no significant differences in the data between groups. For detailed information, refer to Table 1.

**Table 1:** Clinicopathological characteristics.

Variables	Total (n = 157)	Training cohort (n = 108)	External validation cohort (n = 49)	Statistic	*p*
PFS, M (Q_1_, Q_3_)	7.80 (5.13, 11.30)	8.08 (5.07, 10.82)	7.13 (5.60, 12.07)	Z = − 0.10	0.917
Gender, n(%)				χ^2^ = 0.94	0.331
Male	124 (78.98)	83 (76.85)	41 (83.67)		
Female	33 (21.02)	25 (23.15)	8 (16.33)		
Age, n(%)				χ^2^ = 1.91	0.167
< 60	43 (27.39)	26 (24.07)	17 (34.69)		
≥ 60	114 (72.61)	82 (75.93)	32 (65.31)		
BMI, n(%)				χ^2^ = 0.32	0.57
< 25	129 (82.17)	90 (83.33)	39 (79.59)		
≥ 25	28 (17.83)	18 (16.67)	10 (20.41)		
ECOG-PS, n(%)				χ^2^ = 2.41	0.299
0	21 (13.38)	12 (11.11)	9 (18.37)		
1	121 (77.07)	87 (80.56)	34 (69.39)		
2	15 (9.55)	9 (8.33)	6 (12.24)		
Histology, n(%)				–	0.813
Squamous carcinoma	81 (51.59)	54 (50.00)	27 (55.10)		
Adenocarcinoma	74 (47.13)	52 (48.15)	22 (44.90)		
Others	2 (1.27)	2 (1.85)	0 (0.00)		
PD-L1, n(%)				χ^2^ = 1.64	0.44
< 50%	22 (14.01)	16 (14.81)	6 (12.24)		
≥ 50%	27 (17.20)	21 (19.44)	6 (12.24)		
Unknown	108 (68.79)	71 (65.74)	37 (75.51)		
Treatment, n(%)				-	0.448
I	13 (8.28)	10 (9.26)	3 (6.12)		
I + A	15 (9.55)	12 (11.11)	3 (6.12)		
I + C	95 (60.51)	66 (61.11)	29 (59.18)		
I + A + C	34 (21.66)	20 (18.52)	14 (28.57)		
Radiotherapy, n(%)				χ^2^ = 1.63	0.202
No	125 (79.62)	83 (76.85)	42 (85.71)		
Yes	32 (20.38)	25 (23.15)	7 (14.29)		
Number of metastatic, n(%)				χ^2^ = 3.09	0.079
< 2	83 (52.87)	52 (48.15)	31 (63.27)		
≥ 2	74 (47.13)	56 (51.85)	18 (36.73)		
Brain metastatic, n(%)				χ^2^ = 1.52	0.218
No	129 (82.17)	86 (79.63)	43 (87.76)		
Yes	28 (17.83)	22 (20.37)	6 (12.24)		
Liver metastatic, n(%)				χ^2^ = 2.45	0.118
No	130 (82.80)	86 (79.63)	44 (89.80)		
Yes	27 (17.20)	22 (20.37)	5 (10.20)		
Malignant pleural effusion, n(%)				χ^2^ = 2.08	0.15
No	106 (67.52)	69 (63.89)	37 (75.51)		
Yes	51 (32.48)	39 (36.11)	12 (24.49)		
LAR, n(%)				χ^2^ = 0.11	0.736
L-LAR	51 (32.48)	36 (33.33)	15 (30.61)		
H-LAR	106 (67.52)	72 (66.67)	34 (69.39)		
PNI, n(%)				χ^2^ = 0.53	0.467
L-PIV	90 (57.32)	64 (59.26)	26 (53.06)		
H-PIV	67 (42.68)	44 (40.74)	23 (46.94)		
PIV, n(%)				χ^2^ = 0.04	0.838
L-PNI	82 (52.23)	57 (52.78)	25 (51.02)		
H-PNI	75 (47.77)	51 (47.22)	24 (48.98)		

### Relationship between LAR, PIV, PNI, and treatment efficacy

Treatment efficacy was assessed according to the immune-related Response Evaluation Criteria in Solid Tumors (RECIST) version 1.1. Within the training group of 108 participants, the outcomes were as follows: CR was observed in 0 individuals, PR in 17, SD in 24, and PD in 67. This led to a DCR of 37.96% and an ORR of 15.74%. In a separate cohort for validation, comprising 55 individuals, the breakdown was similar: 0 experienced CR, 6 achieved PR, 12 maintained SD, and 31 encountered PD, culminating in a DCR of 36.73% and an ORR of 12.24%.

The association between high and low levels of LAR, PIV, PNI and the DCR and ORR was evaluated using the chi-square test. In the training group, significant statistical differences were found in DCR and ORR when comparing the L-LAR/H-LAR, L-PIV/H-PIV, and L-PNI/H-PNI groups. In the validation group, the Low-PIV/High-PIV group showed statistically significant differences in both DCR and ORR, while the Low-LAR/High-LAR and Low-PNI/High-PNI groups showed significant differences only in the DCR.

Furthermore, in the training group, Patients exhibiting Low-LAR, Low-PIV, and High-PNI demonstrated a markedly improved mPFS in contrast to those presenting with High-LAR, High-PIV, and Low-PNI. In the validation group, similar differences with statistical significance were observed for the Low-PIV/High-PIV groups, as presented in Table 2.

**Table 2:** Treatment response.

Training group	Efficacy				DCR (%)	*p*	ORR (%)	*p*	mPFS (month)	*p*
	CR	PR	SD	PD						
LAR						< 0.001		0.003		0.002
L-LAR	0	11	14	11	69.44		30.56		13.73	
H-LAR	0	6	10	56	22.22		8.33		8.43	
PIV						< 0.001		0.033		< 0.001
L-PIV	0	13	18	26	54.39		22.81		12.83	
H-PIV	0	4	6	41	19.61		7.84		7.70	
PNI						< 0.001		< 0.001		< 0.001
L-PNI	0	3	8	53	17.19		4.69		7.77	
H-PNI	0	14	16	14	68.18		31.82		18.23	

Validation group	Efficacy				DCR (%)	*p*	ORR (%)	*p*	mPFS (month)	*p*
	CR	PR	SD	PD						
LAR						0.025		0.531		0.059
L-LAR	0	3	6	6	60.00		20.00		12.73	
H-LAR	0	3	6	25	26.47		8.82		7.40	
PIV						< 0.001		0.033		< 0.001
L-PIV	0	6	11	8	68.00		24.00		17.83	
H-PIV	0	0	1	23	4.17		0.00		6.63	
PNI						0.007		0.141		0.072
L-PNI	0	1	4	21	19.23		3.85		7.13	
H-PNI	0	5	8	10	56.52		21.74		14.30	

**Figure 1:**
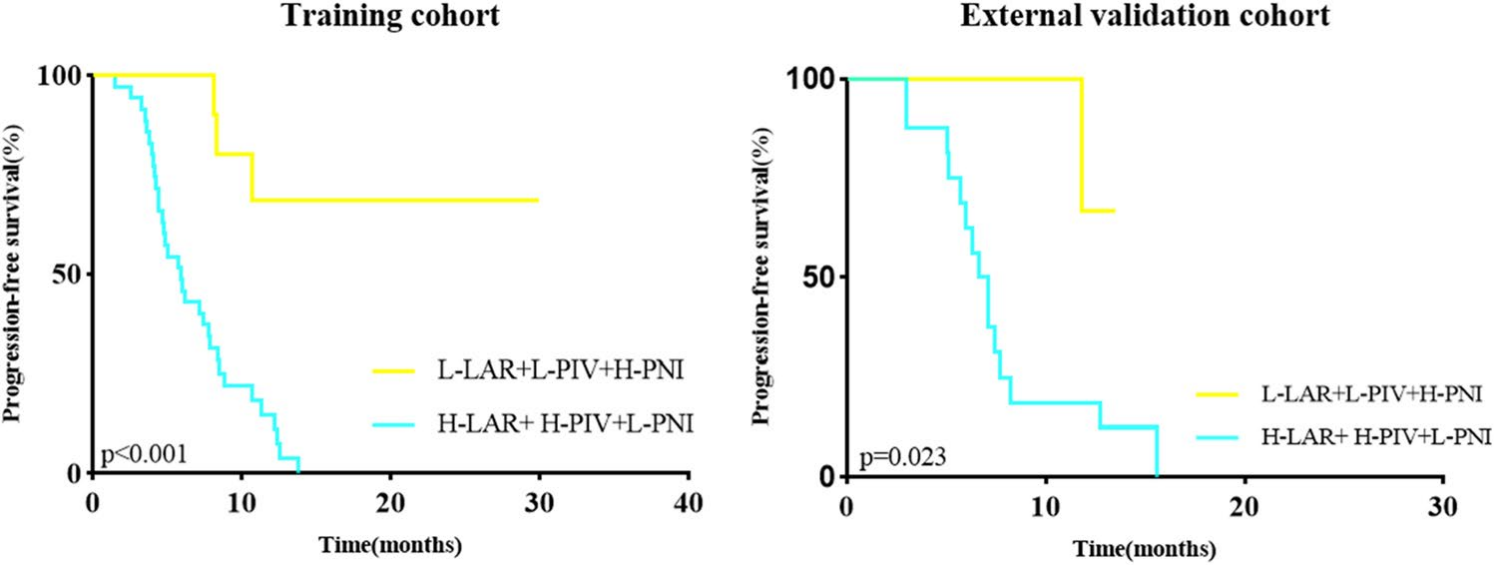
Relationship between LAR, PIV, PNI, and PFS in two groups.

Moreover, when considering the combination of LAR, PIV, and PNI groups, the training group with High-LAR + High-PIV + Low-PNI (n = 35) experienced a reduced PFS compared to the group with Low-LAR + Low-PIV + High-PNI (n = 21), and this difference was statistically significant. The validation group also displayed a similar trend, with High-LAR + High-PIV + Low-PNI (n = 16) versus Low-LAR + Low-PIV + High-PNI (n = 9) showing comparable results to the training group, as depicted in Fig. 1.

### Univariate and multivariate Cox regression analysis

In a training cohort of NSCLC patients, a Cox regression model was employed to evaluate the factors linked to Progression-Free Survival. Univariate analysis indicated that brain metastasis, liver metastasis, malignant pleural effusion, LAR, PNI and PIV were statistically significant and contributed to survival prediction. These significant variables from the univariate analysis were then included in a multivariate analysis, which identified liver metastasis, malignant pleural effusion, PNI, and PIV as risk factors associated with PFS, as shown in Table3.

**Table 3:** COX regression analysis of PFS.

Variables	Univariate analysis		Multivariate analysis	
	HR (95%CI)	*p*	HR (95%CI)	*p*
Gender (Male/Female)	0.90 (0.50–1.63)	0.73		
Age (< 60/≥ 60)	1.35 (0.75–2.46)	0.32		
BMI (< 25/≥ 25)	0.77 (0.42–1.40)	0.39		
ECOG-PS	7.21 (2.88–18.07)	< 0.01	9.43 (3.21–27.68)	< 0.01
Number of metastatic (< 2/≥ 2)	0.78 (0.48–1.27)	0.33		
Brain metastatic (No/Yes)	1.93 (1.17–3.19)	0.01	1.04 (0.59–1.84)	0.882
Liver metastatic (No/Yes)	3.99 (2.08–7.67)	< 0.01	2.70 (1.36–5.37)	< 0.01
Malignant pleural effusion (No/Yes)	1.83 (1.11–3.02)	0.019	0.60 (0.38–0.97)	0.037
Treatment	0.91 (0.70–1.17)	0.449		
Radiotherapy (No/Yes)	0.73 (0.41–1.30)	0.29		
LAR (L-LAR/H-LAR)	2.01 (1.13–3.58)	0.02	1.00 (0.51–1.96)	0.99
PNI (L-PNI/H-PNI)	0.13 (0.06–0.27)	< 0.01	0.11 (0.05–0.25)	< 0.01
PIV (L-PIV/H-PIV)	2.18 (1.34– 3.56)	< 0.01	2.51 (1.38–4.55)	0.01

### Construction and validation of the nomogram

**Figure 2:**
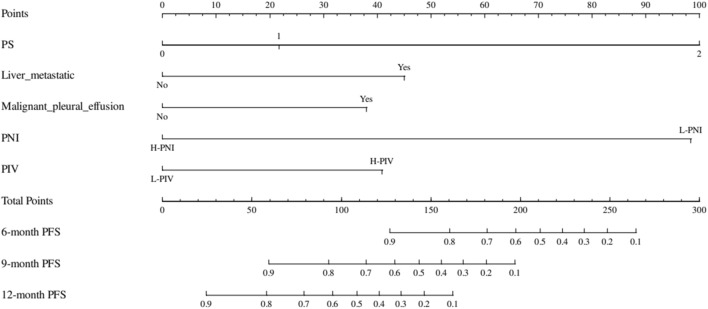
A nomogram for the prediction of probability of PFS at 6, 9, and 12 months.

**Figure 3:**
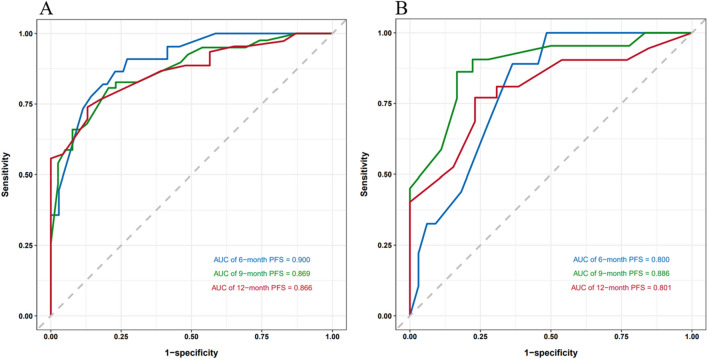
ROC curves for the training group (**A**) and the validation group (**B**) at 6 months, 9 months, and 12 months.

**Figure 4:**
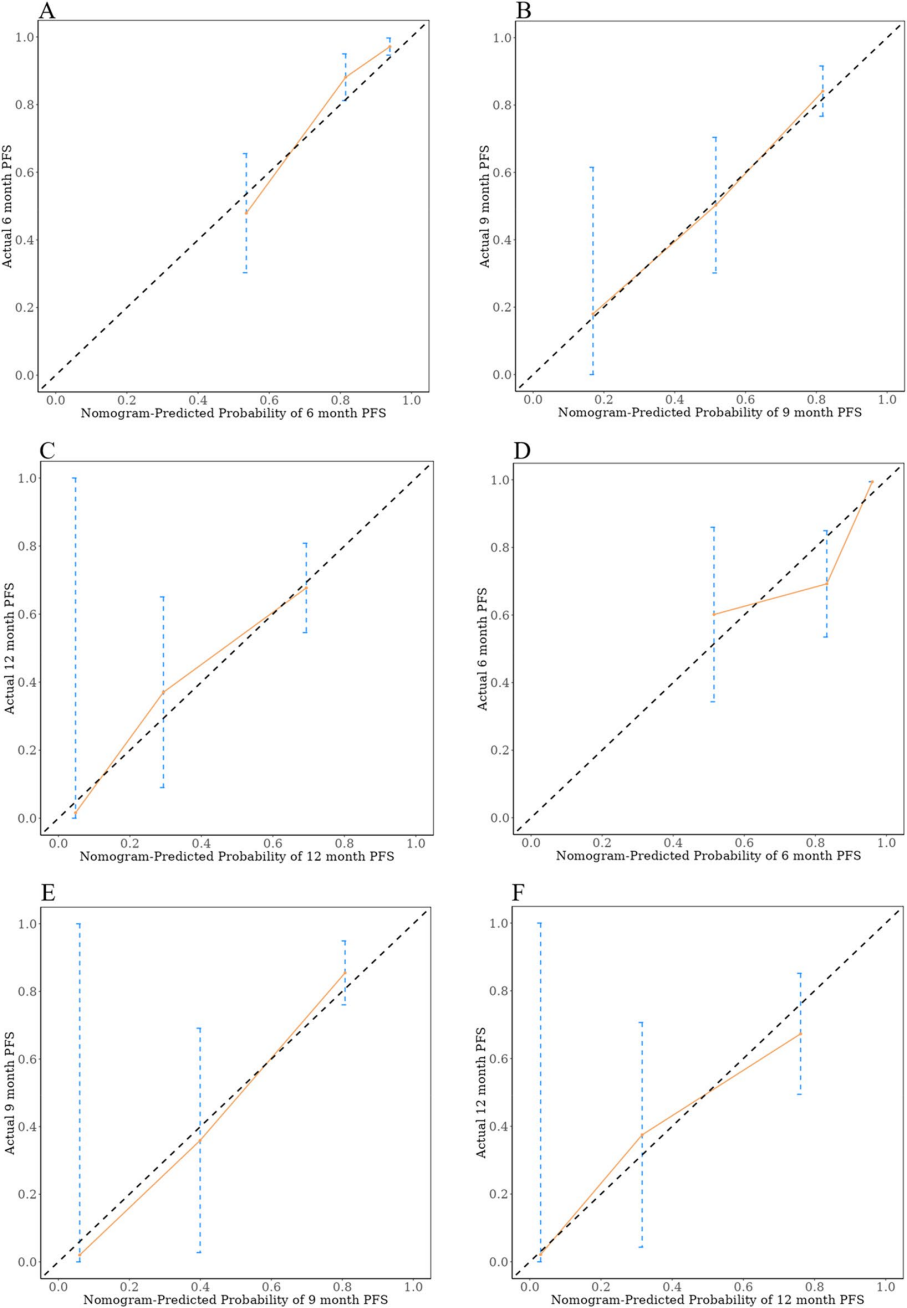
Calibration curves for the probability of PFS at 6 months (**A**), 9 months (**B**), and 12 months (**C**) in the training cohort, as well as at 6 months (**D**), 9 months (**E**), and 12 months (**F**) in the validation cohort.

**Figure 5:**
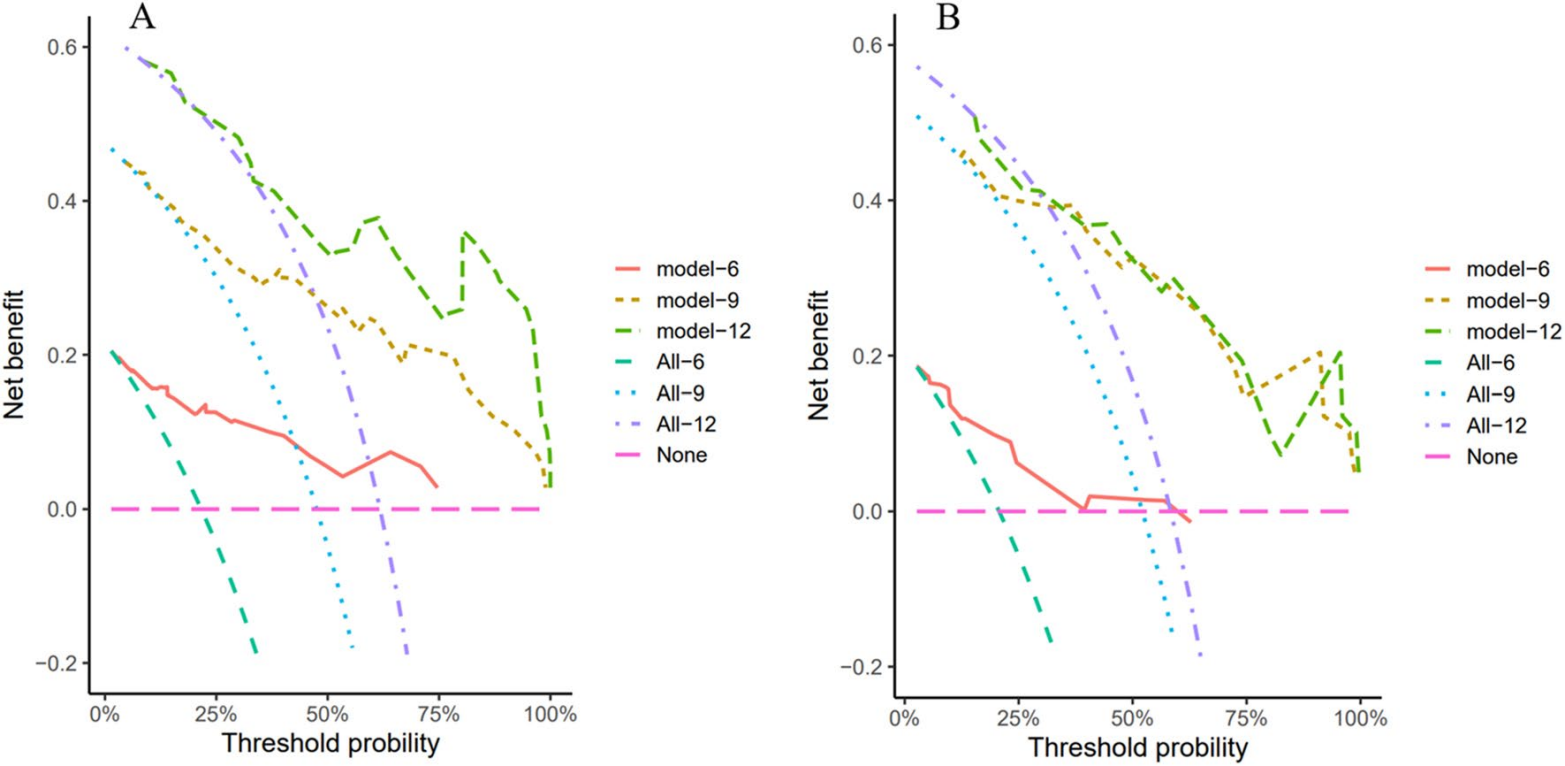
DCA graphs depicting PFS forecasts at intervals of 6, 9, and 12 months were constructed for both the training cohort (**A**) and the validation cohort (**B**).

To further evaluate the predictive capability of PNI, PIV, and other factors on PFS, a nomogram was constructed using the factors identified through multivariate regression analysis in the training cohort. (Fig. 2). Additionally, training cohort exhibited a predictive C-index of 0.78 (95% CI: 0.73–0.84), with AUC values at 6, 9, and 12 months being 0.900, 0.869, and 0.866, respectively (Fig. 3A). Meanwhile, the external validation cohort showed AUCs of 0.800, 0.886, and 0.801 at the corresponding time points (Fig. 3B), indicating good predictive performance. Using the bootstrap resampling method with 500 iterations, the calibration curves drawn for both the training and external validation cohorts demonstrated good consistency between the actual and predicted 6, 9, and 12-month PFS values (Fig. 4). The decision curve analysis presented in Fig. 5 indicates that the risk thresholds for predicting 6, 9, and 12-month PFS in NSCLC patients within the modeling group ranged from 0.01 to 0.90, 0.05–0.98, and 0.08–0.99, respectively. Similarly, the risk thresholds in the external validation set for forecasting PFS at 6, 9, and 12 months were 0.03–0.63, 0.12–0.97, and 0.15–0.98, respectively. The wide range of threshold values in the DCA curves suggests a substantial net benefit and indicates that the prediction model has good clinical applicability.

## Discussion

The presence and progression of tumors are intimately linked to the body's inflammatory response. Chronic inflammation can reshape the extracellular matrix and promote angiogenesis and lymphangiogenesis within the tumor microenvironment, thereby facilitating tumor growth and metastasis^13^. Lymphocytes, neutrophils, monocytes, and platelets are key components of the inflammatory response, playing a crucial role not only in host defense but also in tumor biology. Lymphocytes can regulate their own immune response to directly recognize and kill tumor cells, inhibiting tumor proliferation and migration. They can also induce apoptosis in tumor cells through the Fas/FasL pathway^14^, playing an important role in tumor immune surveillance. Neutrophils can reflect local or systemic inflammatory activity and, by releasing various inflammatory mediators and cytokines such as tumor necrosis factor-alpha (TNF-α) and vascular endothelial growth factor (VEGF), can suppress lymphocyte-mediated anti-tumor effects, thus promoting tumor invasion and metastasis^15^. Monocytes that migrate to the tumor tissue can differentiate into tumor-associated macrophages, releasing growth factors that inhibit cytotoxic T lymphocytes and natural killer cells, facilitating immune escape^16,17^. They can also induce epithelial-mesenchymal transition in tumor cells, affecting the penetration of chemotherapy drugs and contributing to chemoresistance^18^. Platelets can bind to tumor cells and secrete factors like TGF-β, inducing epithelial-mesenchymal transition in tumor cells and increasing their migratory potential. Additionally, tumor cells can stimulate platelet aggregation to form thrombi, helping the tumor cells evade immune surveillance^19,20^. The Peripheral Inflammatory Value is a biomarker derived from platelets, neutrophils, monocytes, and lymphocytes. High baseline levels suggest a heightened inflammatory response, lower immunity, and a greater likelihood of tumor progression. The study's Cox analysis supports the association between high PIV and increased risk of advanced NSCLC progression, as evidenced by the H-PIV group exhibiting lower disease control rates and shorter mPFS compared to the L-PIV group. Similar results have also been confirmed in the research conducted by Zeng R and others^21^.

Serum albumin serves as an indicator of both the body’s nutritional status and the host's immune and inflammatory responses. Certain inflammatory cytokines can inhibit the synthesis of albumin, and oxidative stress during tumor progression may lead to albumin denaturation. These factors lead to a swift decline in serum albumin levels among cancer patients^22,23^. Additionally, the binding of drugs to serum albumin can affect their half-life and thus impact therapeutic efficacy. The Prognostic Nutritional Index (PNI) is a cumulative value of albumin levels and lymphocyte count, reflecting the host’s immune status and nutritional condition. The results of this study indicate that the high PNI group had better disease control rates and median progression-free survival compared to the low PNI group. Cox analysis suggested that L-PNI is a risk factor for tumor progression. Similarly, in a study of NSCLC patients receiving first-line pembrolizumab treatment^24^, researchers found that patients with a high PNI had significantly better PFS than those with a low PNI, which aligns with the findings of this study. Furthermore, under aerobic conditions, metabolically active tumor cells still require anaerobic glycolysis to obtain energy, a phenomenon known as the Warburg effect. This process necessitates the upregulation of lactate dehydrogenase (LDH)^25^; the lactate accumulated through anaerobic glycolysis can promote tumor metastasis by upregulating vascular endothelial growth factor through hypoxia-inducible factor 1-alpha (HIF-1α)^26^. It can also inhibit the surveillance of T cells and NK cells on tumors^27^. The Lactate Dehydrogenase to Albumin Ratio (LAR) has been validated in multiple studies as a potential predictive indicator for immunotherapy in NSCLC^7,28,29^, with low baseline levels of LDH being significantly correlated with extended PFS. In this study, although the multivariate analysis of LAR was not statistically significant, the low LAR group showed better disease control rates, response rates, and median PFS compared to the high LAR group, presenting similar outcomes.

Malignant pleural effusion frequently occurs in the advanced stages of lung cancer, often resulting from increased capillary permeability due to tumor invasion of the pleural surface or from obstruction of lymphatic fluid return caused by tumor invasion into the mediastinal lymphatic system. Research has indicated that malignant pleural effusion is a predictive factor for reduced PFS in NSCLC patients receiving first-line pembrolizumab or second-line nivolumab treatment^30,31^, which may be related to the relatively higher levels of VEGF in the blood plasma of patients with malignant pleural effusion. VEGF can promote tumor proliferation and metastasis and also induce immune suppression^32^. The brain, a common site for NSCLC metastasis, has traditionally been considered an “immune-privileged” organ due to the blood–brain barrier, which reduces the availability of drugs within the brain and hinders the entry of lymphocytes^33^, thereby inhibiting the effectiveness of immune checkpoint inhibitors. In comparison, liver metastasis is often underestimated. Recent meta-analyses have also assessed the relationship between the presence of liver metastasis and the effectiveness of ICIs in NSCLC patients, finding that patients without liver metastasis can benefit more significantly from immunotherapy^34^. Liu et al.^35^ observed similar results, noting a decrease in the short-term efficacy of ICIs and a reduction in PFS in NSCLC patients with liver metastasis. The related mechanism might involve the liver’s immunosuppressive microenvironment, which may facilitate tumor cell evasion from antitumor immune surveillance induced by immunotherapy, thus reducing the effectiveness of the treatment^36^. Hence, the combination of angiogenesis inhibitors could enhance the tumor microenvironment in liver metastasis, potentially improving the effectiveness of immunotherapy^37^.

In summary, peripheral blood markers such as LAR, PIV, and PNI can be calculated from routine blood test data, offering advantages such as safety, low cost, and easy accessibility, and they hold certain significance in guiding the effectiveness of ICIs in late-stage NSCLC. This article introduces a Nomogram tool, crafted through variable selection via Cox regression analysis and has conducted external validation of the predictive model, suggesting that the established predictive model is reliable. The model's clinical feasibility is further demonstrated by DCA. Nonetheless, the retrospective design of the study introduces potential biases related to diverse treatment plans, prior therapies, and various response metrics, pointing to the necessity for future prospective research across multiple centers with more extensive cohorts. The study's limited follow-up scope means it only addresses the PFS of patients with advanced NSCLC treated with immunotherapy. Moreover, the absence of other critical predictive markers like Tumor-Infiltrating Lymphocytes (TILs), Tumor Mutational Burden (TMB), and Human Leukocyte Antigen (HLA) indicates room for broader investigative work.

## Conclusion

In essence, the developed Nomogram, incorporating a range of factors, demonstrates respectable accuracy and the capability to distinguish outcomes, thus serving as a tool for estimating the likelihood of PFS in advanced NSCLC patients receiving immunotherapy. This aids healthcare professionals in making informed therapeutic choices. Despite these advancements, there is a clear need for further research with larger cohorts, prospective designs, and external validations to refine and enhance the predictive value of Nomograms for this patient group.

## Data Availability

All data generated or analyzed during this study are included in this published article. The data that support the findings of this study are available from the corresponding author, Weirong Yao, upon reasonable request.

## Abbreviations

NSCLC: Non-small cell lung cancer
ICIs: Immune checkpoint inhibitors
PD-1/PD-L1: Programmed cell death receptor 1 and its ligand
irAEs: Immune-related adverse events
PIV: Pan-immune-inflammation index
PNI: Prognostic nutritional index
LAR: Lactate dehydrogenase to albumin ratio
CP: Complete response
PR: Partial response
SD: Stable disease
PD: Progressive disease
ORR: Objective response rate
DCR: Disease control rate
AUC: Area under the ROC curve
TNF-α: Tumor necrosis factor-alpha
VEGF: Vascular endothelial growth factor
TILs: Tumor-infiltrating lymphocytes
TMB: Tumor mutational burden
HLA: Human leukocyte antigen
