# Author Correction: Predictive value of inflammation and nutritional index in immunotherapy for stage IV non-small cell lung cancer and model construction

**DOI:** 10.1038/s41598-024-70611-3

**Published:** 2024-08-22

**Authors:** Wenqian Lei, Wei Wang, Shixiang Qin, Weirong Yao

**Affiliations:** 1 Graduate School, Jiangxi Medical College, Nanchang University, 461 Bayi Avenue, Donghu District, Nanchang, 330006 Jiangxi China; 2 Department of Oncology, Jiangxi Provincial People’s Hospital, 152 Aiguo Road, Nanchan, 330006 Jiangxi China; 3 Postgraduate training base Alliance of Wenzhou Medical University (Zhejiang Cancer Hospital), Wenzhou Medical University, 270 Xueyuanxi Road, Lucheng District, Wenzhou, 325027 Zhejiang China; 4 Graduate School, Bengbu Medical University, Bengbu, 233030 Anhui China

Correction to: *Scientific Reports* 10.1038/s41598-024-66813-4, published online 30 July 2024

The original version of this Article contained an error in the Materials and methods, under the subheading ‘Ethics approval and consent to participate’. As a result,

“The study was approved by the Medical Ethics Committee of Jiangxi Provincial People’s Hospital. All methods were carried out in accordance with relevant guidelines and regulations. All methods were carried out in accordance with declaration of Helsinki. Informed consent was waived by the Jiangxi Provincial People’s Hospital due to the retrospective nature of this study.”

now reads:

“The study was approved by the Medical Ethics Committee of Jiangxi Provincial People’s Hospital. All methods were carried out in accordance with relevant guidelines and regulations. All methods were carried out in accordance with declaration of Helsinki. Informed consent was waived by the Jiangxi Provincial People’s Hospital due to the retrospective nature of this study. Ethical Approval ID: V1.0,20240209.”

The original Article has been corrected.

